# Effect of Marine Microalgae Biomass (*Nannochloropsis gaditana* and *Thalassiosira* sp.) on Germination and Vigor on Bean (*Phaseolus vulgaris* L.) Seeds “Higuera”

**DOI:** 10.3390/life15030386

**Published:** 2025-02-28

**Authors:** Brisia Lizbeth Puente-Padilla, Gabriel Ivan Romero-Villegas, Alberto Sánchez-Estrada, Luis Alberto Cira-Chávez, María I. Estrada-Alvarado

**Affiliations:** 1 Departamento de Biotecnología y Ciencias Alimentarias, Instituto Tecnológico de Sonora, Calle 5 de Febrero 818, Ciudad Obregón 85000, Sonora, Mexico; brisiapuente@hotmail.com (B.L.P.-P.); luis.cira@itson.edu.mx (L.A.C.-C.); 2 Beacon Development Department, King Abdullah University of Science and Technology, Thuwal 23955, Saudi Arabia; 3 Coordinación de Tecnología de Alimentos de Origen Vegetal, Centro de Investigación en Alimentación y Desarrollo, A.C. (CIAD, A.C.), Gustavo Enrique Astiazarán Rosas 46, Hermosillo 83304, Sonora, Mexico; aestrada@ciad.mx

**Keywords:** microalgae biomass, biostimulant, sustainable agriculture, bioactive molecules

## Abstract

The production of marine microalgae provides a sustainable solution for agriculture, acting as biostimulants to enhance seed germination, vigor, and early growth. In the present work, the parameters of pH, airflow, and dilution speed were established to produce biomass of two species of marine algae (*Nannochloropsis gaditana* and *Thalassiosira* sp.); in addition, its capacity to stimulate the germination of bean seeds was evaluated. The experimental treatments included three biomass concentrations (C_b_) of both microalgae species (0.5, 1, and 1.5 g·L^−1^) and a control (distilled water) at two temperatures (25 and 35 °C). The rate, index, average time, time at 50% germination, and vigor were evaluated. The results indicated that the highest yield of microalgae biomass was obtained with D = 0.3 day^−1^ for *N. gaditana* and 0.2 day^−1^ for *Thalassiosira* sp. Microalgae biomass showed activity as a biostimulant on germination, improving the germination rate and reducing the germination time with better vigor for the seedlings at each of the evaluated concentrations.

## 1. Introduction

Microalgae are microscopic, photosynthetic, and aquatic organisms of great interest due to their potential use in agriculture [1,2]. Among the vast array of microalgae species, *Nannochloropsis gaditana* (Eustigmatophyceae) and *Thalassiosira* sp. (Diatoms) have attracted interest in the biotechnology and nanotechnology sectors applied to agriculture [3]. These species are strong candidates due to their adaptability, notable biomass production, and secretion of inorganic and organic compounds, including silica, growth-promoting hormones, and bioactive molecules that can positively impact plant growth [4].

The large-scale production of microalgae requires the proper selection of the cultivation medium, environmental conditions, and harvesting methods needed to achieve the highest biomass productivity (P_b_) at the lowest production and investment costs [5,6]. Extensive research is ongoing to explore different environments, including seawater, freshwater, and even polluted water, in addition to optimizing CO_2_ and photoperiod requirements to increase biomass yields and enhance beneficial microalgal compounds [7].

The pH of the culture medium influences both the proportion of species and the chemical form of certain nutrients [8]. Each microalga is adapted to a specific pH range that allows optimal enzymatic processes within the microorganism [9], and pH can be regulated by injecting CO_2_ or adding an acid or a base [10].

Microalgae cultivated under neutral pH typically uptake and fix soluble CO₂ in the aqueous growth medium. However, some salt precipitation (CO_3_^2−^, OH^−^, and PO_4_^3−^) may occur, leading to the chemical deterioration of the medium and potential cell damage [10]. While a high CO_2_ concentration (~5%, *v*·*v*^−1^) can increase P_b_, the continuous supply of CO_2_ lowers the pH of the medium, affecting microalgae physiology and cell growth and increasing the risk of contamination, which ultimately reduces biomass production [11,12].

Another critical factor in microalgae cultures is agitation, which ensures a homogeneous distribution of nutrients within the reactor, improves light reception by cells, and prevents cell sedimentation at the reactor’s bottom [13]. This factor is essential to control because stagnant conditions often lead to excessive dissolved oxygen saturation, which can inhibit photosynthesis and photorespiration in microalgae [14]. Consequently, mass cultures of microalgae are generally grown in CO_2_:O_2_ ratios much higher than in the air [15]. A study [16] reported that an airflow rate of 2 L min^−1^ was optimal for *N. gaditana*, as it promoted continuous cell movement and increased biomass production.

The dilution rate (D) also plays a crucial role in biomass production. The medium flow rate determines the nutrient supply rate and directly impacts microorganism growth rates. However, the optimal dilution rate depends on the specific species, water quality, and cultivation medium, meaning each culture condition requires an individualized dilution rate [17].

Microalgal biomass contains bioactive molecules with functions similar to those found in land plants [18,19]. Some of these molecules, such as auxins, abscisic acid, cytokinins, ethylene, and gibberellins, act as organic biostimulants [20]. For example, the oceanic microalga *Nannochloropsis* spp. has demonstrated endogenous abscisic acid and cytokinin activity, producing physiological effects comparable to those of higher plants [21].

The germination process plays a fundamental role in a plant’s life cycle, as successful germination is crucial for seedling establishment, growth, and crop yields [22]. Seed germination marks the transition from dormancy to active growth, involving several physiological and biochemical mechanisms. These mechanisms can be stimulated using nutrient-rich compounds, growth regulators, and enzymes to enhance germination rates and improve seed vigor [23].

Microalgae biomass can positively influence seed germination, root growth, and overall development [23]. Recent studies have shown that microalgal extracts, rich in bioactive compounds, can enhance germination speed, vigor, and overall seedling growth [24], benefiting both easy- and hard-to-germinate seeds. Utilizing microalgal biomass to promote seed germination represents an innovative and sustainable approach to modern agriculture [25]. However, its full agricultural potential remains relatively unexplored.

Bean seeds typically exhibit high germination rates [26]; however, these rates can decline significantly under suboptimal temperatures [27]. Beans are cultivated in the Yaqui Valley region (Ciudad Obregón, Sonora, Mexico; 27.4678, −109.98511) [27] and in other areas where temperatures exceed 30 °C [28]. Natural biostimulants derived from microalgal biomass may help restore seed viability and protect against abiotic stress [23].

This study aims to optimize pH, airflow, and dilution rate conditions to maximize the biomass production of *Nannochloropsis gaditana* and *Thalassiosira* sp. for agricultural applications. Additionally, this study seeks to demonstrate that marine microalgal biomass can serve as a biostimulant to enhance the germination of “Higuera” bean seeds.

## 2. Materials and Methods

### 2.1. Strains and Growing Conditions

**Figure 1:**
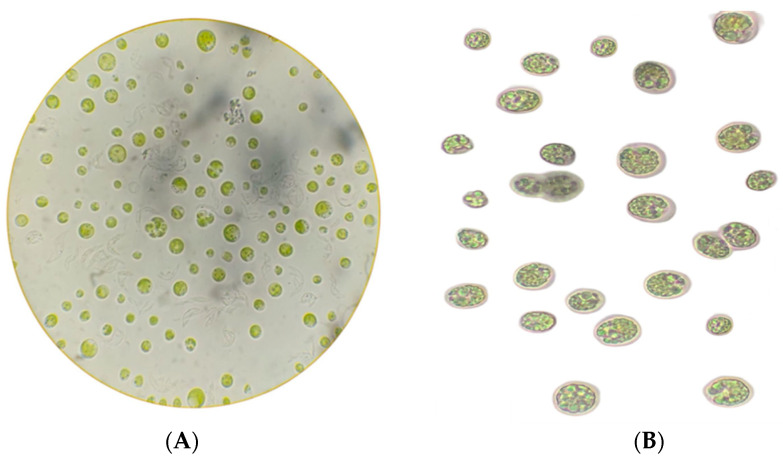
Microalgae species (**A**) *Nannochloropsis gaditana* and (**B**) *Thalassiosira* sp.

The marine microalgae *Nannochloropsis gaditana* and *Thalassiosira* sp. were donated by the *Instituto de Acuacultura del Estado Sonora* (Sonora State Aquaculture Institute, Hermosillo, Sonora, México) (Figure 1). They were grown under photoautotrophic conditions. The inoculum cultures were maintained photoautotrophically in 0.25 L Erlenmeyer flasks containing 170 mL of f/2 culture medium. The microalgae were grown aseptically at 25 °C, with an aeration rate of 0.6 *v*·*v*^−1^·min^−1^ and under continuous illumination at 250 μmol·m^−2^·s^−1^ (Plusrite T8).

Microalgae cultures were carried out in triplicate using an f/2 culture medium [29]. Macro- and micronutrients were prepared in sterilized water via autoclaving (Brinkmann 2340M, Santa Rosa, CA, USA) at 121 °C for 15 min. For each experiment, 2.9 L of polypropylene photobioreactors were equipped with an inlet valve and a discharge valve (harvest). Air was injected from the reactor base to ensure proper mixing. A pH sensor (HI 1131B Hanna; Cluj, Romania) was placed at the top of the reactor, and pH was maintained by injecting pure CO₂ on demand at 1.0 L·min^−1^. The reactors were illuminated using 32 W high-efficiency fluorescent lamps (Plusrite T8) at 300 µE·m^−2^·s^−1^ irradiance (12 h dark: 12 h Light). The average irradiance value was measured using a spherical sensor (QSPL2100; San Diego, CA, USA) with an algal medium (Table 1) and artificial seawater containing 3.2% NaCl.

### 2.2. Treatments

pH, airflow, and D were analyzed independently to determine the ideal growing conditions for achieving higher P_b_. In the first experiment, airflow was measured at a neutral pH (7), and five airflow rates (0.5, 1.0, 1.5, 2.0, and 2.5 L min^−1^) were evaluated. Subsequently, pH was assessed by testing different levels (6.5, 7.0, 7.5, 8.0, and 8.5). Finally, dilution rates (0.1, 0.2, 0.3, 0.4, and 0.5 day^−1^) were tested and adjusted based on the optimal pH and airflow level determined from biomass production in the experiment.

### 2.3. Analytical Procedures

C_b_ was determined daily in each experiment by measuring absorbance at 560 and 620 nm using a spectrophotometer (Thermo Scientific Multiskan Go, Vantaa, Finland). The spectrophotometric measurements were verified through dry weight determinations twice a week.

Dry weight, C_b_, was measured by centrifuging 100 mL of culture for 10 min at 12,000× *g*. The sediment was then collected and freeze-dried in a freeze-dryer (Yamato DC401, Japan) for 24 h before being weighed and later used for germination experiments. P_b_ was calculated by multiplying C_b_ by the set dilution rate (D) using Equation (1).

(1) $$P_{b}=C_{b}\cdot D$$

### 2.4. Determination of Lipids, Proteins, Carbohydrates, and Ashes

The microalgae biomass lipid fraction was determined through the gravimetric method for extracts obtained with chloroform–methanol [30]. Approximately 100 mg of biomass was taken from the dry (lyophilized) sample, and 2 mL of chloroform–methanol 2:1 (*v*·*v*^−1^) was added and stirred. It was centrifuged at 9000× *g* for 5 min (ST8R Sorvall, Thermo Sci, Heiligen, Germany). The liquid phase was separated from the biomass, and the washing was repeated with a chloroform–methanol ratio of 2:1 until the biomass was white. Three mL of 0.1 N HCl and 0.3 mL of 0.5% MgCl_2_ were added to separate the proteins. It was shaken and centrifuged. The black phase was taken with a Pasteur pipette, transferred to a previously weighed test tube, and placed in a thermoblock to evaporate the solvent until a constant weight was reached.

Soluble proteins were analyzed via the Lowry method [31], which uses bovine serum albumin as the standard protein. Appropriate solvent and reagent blanks were used for every assay, and readings were taken at 750 nm.

Total ashes were determined from freeze-dried microalgae biomass with a gravimetric method [32]; 0.200 g of biomass was weighed and incinerated in a muffle (Terlab TE-M12D, Jalisco, Mexico) at 550 °C for 24 h. The difference in weights calculated the total ash. The carbohydrate percentage was calculated according to Equation (2), considering lyophilized biomass:

CH = 100% − (Proteins + Lipids + Ashes) (2)

### 2.5. Germination Assays

The “Higuera” bean variety was selected for this study due to its agronomic relevance, high quality, and adaption to the edaphic conditions of the region (27.4678, −109.98511); it is also highly accepted on the market and rich in bioactive compounds bioactive properties [33], making it an ideal candidate for evaluating the effectiveness of microalgal biostimulants in enhancing germination and seedling vigor. The treatments consisted of 4 treatments with different dry-weighted microalgae biomass concentrations (C_b_) 0.0 (control), 0.5 (T1), 1.0 (T2), and 1.5 (T3) g of freeze-dried microalgae biomass L^−1^ of distilled water, using both microalgae strains separately, and two temperatures were tested (25 and 35 °C).

**Figure 2:**
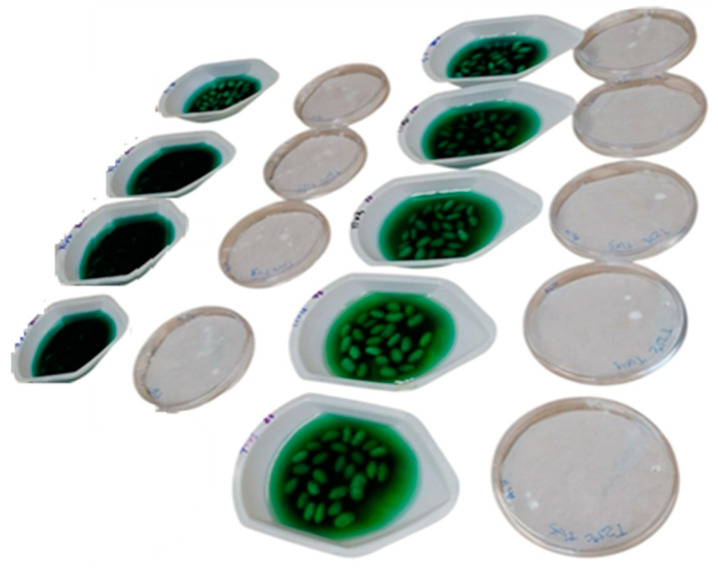
Bean seeds treated with different biomass concentrations of two microalgae species (*Nannochloropsis gaditana* and *Thalassiosira* sp.).

According to the International Seed Testing Association, bean seeds were grouped into four groups of 100 per tray [34]. The seeds were imbibed for 15 min using 3 different C_b_ suspended in distilled water as a reference for each microalgae strain and placed and placed in Petri dishes with moist absorbent paper for germination (Figure 2). Consequently, germination was determined by counting germinated seeds every 12 h from the first germinated seed until 92 h.

To evaluate the weight increase, 10 seeds were taken from each tray for all treatments (at 96 h). The germination mean time (GMT) was evaluated according to Equation (3). where “ti” is the start time of first germinating (hours), “t” is the time of emerging seeds in any counting period (hours), and “n“ is the number of non-accumulated germinated seeds counted in any counting period germination mean time (GMT) [35].

GMT = ti + [(Σn∙t)/Σn] (3)

T50 (the time in which 50% of the total seed germinates) was determined according to Equation (4) [36], where N is the final number of germinated seeds, nᵢ is the initial number of germinated seeds, nⱼ is the accumulated final number of germinated seeds, tᵢ is the initial germination time, and tⱼ is the final germination time. The germination index (GI) is the ratio between the number of germinated seeds and the observed time, determined according to Equation (5) [37]. The plant vigor index (SV) components (SVI, SVII, and SVIII) are determined according to Equations (6)–(8) [38].

(4) $$T_{50}=t_{i}+[((N/2-n_{i}))/((n_{j}-n_{i}))]\times (t_{j}-t_{i})$$

(5) $$GI=\frac{\#germinated\;seeds}{ti}+\ldots \frac{\#germinated\;seeds}{t\;of\;n\;counting}\ldots +\frac{\#germinated\;seeds}{tf}$$

(6) SVI=root length(cm)×%GF

(7) $$SVII=root\;length\left(cm\right)+shoot\;length(cm)\times \%GF$$

(8) SVI=Seedling Dry Weight×%GF

The final germination percentage represents the total number of germinated seeds. It is calculated according to Equation (9), where N is the number of germinated seeds, and SS is the total number of seeds per assay. It is expressed as a percentage.

(9) $$\%GF=\left(\frac{N}{SS}\right)\times 100$$

### 2.6. Glucose

Glucose was quantified in non-germinated (prior to germination) and germinated bean seeds after 72 h of imbibition [39]. A 1.0 g sample of lyophilized bean seeds (from a pool of 10 no-geminated or germinated seeds depending on treatment and time) was taken and degreased using hexane (Sigma-Aldrich, Steinheim, Germany). The mixture was homogenized for 2 min using an Ultra-Turrax T25 homogenizer (IKA, Staufen, Germany) coupled with a dispersion shaft (10 G, IKA, Staufen, Germany) in a 10 mL water–ethanol solution (10:10 *v*/*v*). Subsequently, the mixture was heated in a water bath at 100 °C for 15 min.

The samples were then filtered, and 2 mL of the filtrate was transferred to microtubes (Eppendorf) and centrifuged at 3700× *g* for 15 min at room temperature. The supernatant was filtered using a 0.22 µm GV-type filter (Millipore). Finally, 20 µL of the filtered supernatant was injected into HPLC equipment equipped with a refractive index detector and a Supelco LC-NH_2_ column (250 mm in length, 4.6 mm inner diameter, and 5 µm particle size) (Sigma Chemical Co., St. Louis, MO, USA), along with an LC-NH_2_ guard column.

For the mobile phase, a mixture of acetonitrile and water (80:20 *v*/*v*) was used, with a flow rate of 1 mL·min^−1^ for 15 min. Detection and quantification were performed using glucose HPLC-grade (Sigma-Aldrich) calibration curves at different concentrations, expressed in mg·g^−1^ of dry weight.

### 2.7. Amylose

Amylose was quantified in germinated and non-germinated bean seeds 72 h after the imbibition treatment [40,41]. The seeds were freeze-dried, degreased with hexane, and ground. A 1.0 g sample of ground seeds was mixed with 10 mL of 6M urea (JT Baker, Phillipsburg, NJ, USA), followed by adding 2.0 mL of DMSA (1:9 *v*/*v*, Sigma-Aldrich, St Louis, MO, USA). The mixture was placed in a water bath at 100 °C for one hour, shaking every 10 min. After cooling, the samples were centrifuged at 3700× *g*, and 0.5 mL of the supernatant was mixed with 25 mL of distilled water in a tube. Subsequently, 1 mL of I_2_/KI solution (2 mg of I_2_/20 mg KI, Sigma-Aldrich, St Louis, MO, USA) was added, and the absorbance was measured in a spectrophotometer (Hach 6300, Berlín, Germany) at 730 nm. Quantification was performed using an amylose standard curve mixture (Sigma-Aldrich, St Louis, MO, USA). The R^2^ value for the amylose standard curve was 0.9921. Apparent amylose content was expressed as a percentage (%).

### 2.8. Statistical Analysis

An analysis of variance (ANOVA) was performed based on a completely randomized experimental design with five treatments and three repetitions in each experiment for microalgae biomass with a total of 30 experimental units. The germination experiments had four treatments and four repetitions; each repetition consisted of 100 seeds, and the mean comparison test was conducted using the Tukey test (*p* ≤ 0.05) with the statistical package Statgraphics Centurion XVI (StatPoint Technologies, Inc., Warrenton, VA, USA).

## 3. Results

**Figure 3:**
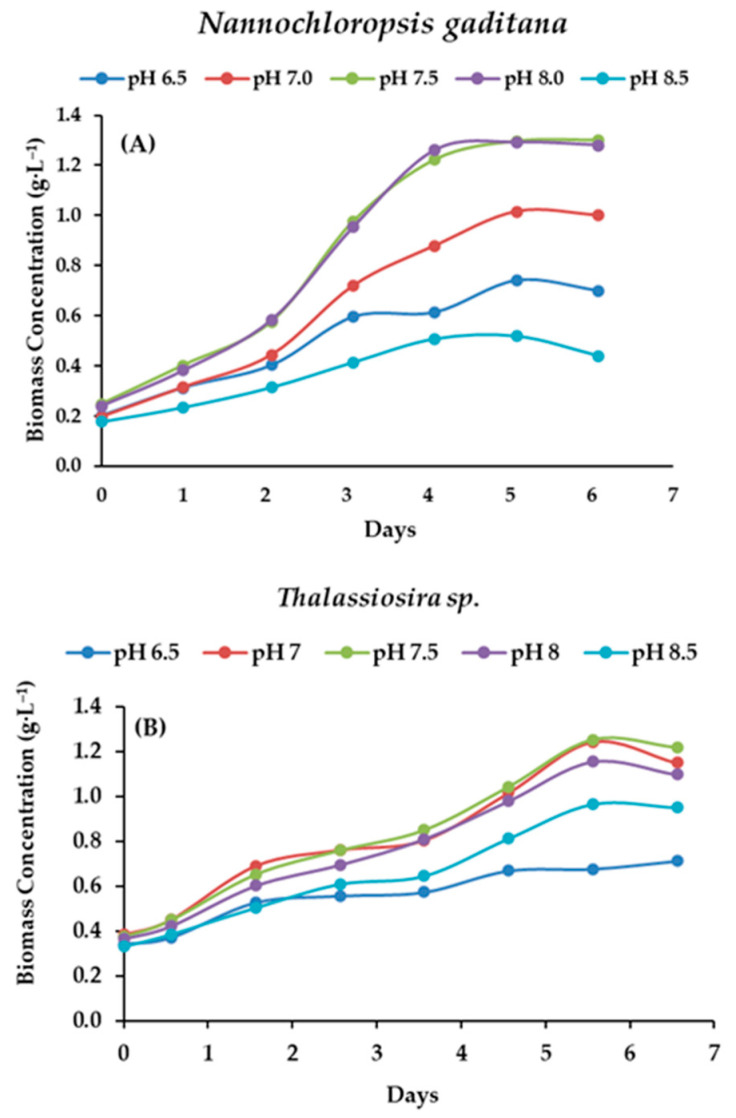
Influence of pH in the growth curve of (**A**) *Nannochloropsis gaditana* and (**B**) *Thalassiosira* sp.

According to the results for C_b_ at different pH levels (Experiment 1, Figure 3), there were significant differences in C_b_ among the different pH levels. *N. gaditana* and *Thalassiosira* sp. achieved the highest C_b_ values at pH 7.5–8, with 1.3 g·L^−1^ and 1.25 g·L^−1^, respectively. In contrast, at pH 6.5 and 8.5, *Thalassiosira* sp. showed lower values (0.71 g·L^−1^ and 0.50 g·L^−1^, respectively), while *N. gaditana* recorded 0.71 g·L^−1^ and 0.97 g·L^−1^, respectively. A similar pH range is used for most of the microalgae; e.g., the C_b_ value in *Thalassiosira weissflogii* is 35–50% higher at a pH range of 7.80–7.85 when grown in f/2-Si medium [42,43,44].

**Figure 4:**
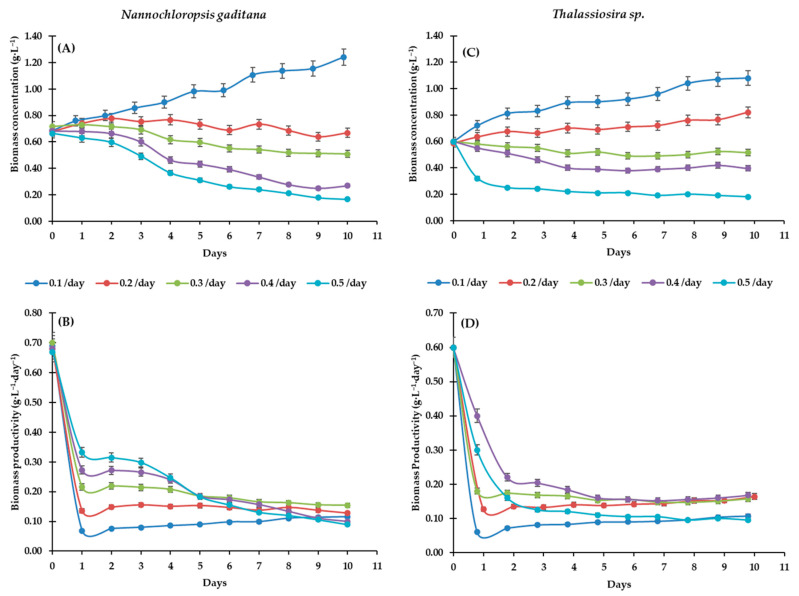
Influence of the dilution rate the biomass concentration and biomass productivity of *Nannochloropsis gaditana* and *Thalassiosira* sp. (**A**) C_b_ of *Nannochloropsis gaditana*, (**B**) P_b_ of *Nannochloropsis gaditana*, (**C**) C_b_ of *Thalassiosira* sp., and (**D**) P_b_ of *Thalassiosira*.

In the dilution rate (D) strain assays (Figure 4), pH was adjusted to 7.5, as this condition yielded the highest C_b_ in Experiment 1. The optimum dilution rate (D) was determined for each microalgae strain culture. D of 0.10 day^−1^ resulted in the highest C_b_ for *Thalassiosira* sp. (0.88 g·L^−1^) and a P_b_ of 0.09 g·L^−1^·day^−1^, outperforming other treatments. However, the maximum C_b_ was observed at D = 0.30 day^−1^, though it was not statistically significant (*p* < 0.05) compared to 0.2, 0.4, and 0.5 day^−1^; it was significantly higher than at D = 0.1 day^−1^. A similar trend was observed for *N. gaditana*, with C_b_ = 1.06 g·L^−1^ and P_b_ = 0.175 g·L^−1^·day^−1^. Cultures of this microalga exhibited maximum C_b_ values of 0.88 g·L^−1^ and P_b_ of 0.16 g·L^−1^·day^−1^, while the lowest concentrations were recorded at D = 0.5 day^−1^ (0.32 g·L^−1^).

### 3.1. Germination Mean Time, T50 Germination, Germination Final Percentage, Germination Index, and Vigor Index

The biomass of two microalgae species was evaluated for dose-response effects on GMT, T50, GF%, GI, SVI, SVII, and SVIII. GMT values in bean seeds treated with *Thalassiosira* sp. biomass showed no significant differences (*p* < 0.05) at 25 °C. However, at 35 °C, T1 and T2 treatments exhibited significant differences (*p* < 0.05) compared to the control. The reduction in GMT ranged between 20 and 25 h in the microalgae biomass treatments compared to the control. In contrast, *N. gaditana* treatments resulted in significant differences (*p* < 0.05) in GMT, with T1 and T2 reducing germination time by 10–15 h at 35 °C compared to the control (Table 2).

T50 results (the time at which 50% of the total seeds germinated) are presented in Table 2. T1, T2, and T3 treatments with *Thalassiosira* sp. microalgae reduced T_50_ by 2–5 h at 25 °C and up to 10 h at 35 °C compared to the control, which recorded 65.23 and 78.39 h, respectively. Similarly, in *N. gaditana* treated seeds, T_50_ was reduced by 4, 6, and 10 h at 35 °C compared to the control.

Treatments with *N. gaditana* biomass achieved maximum germination frequency (GF%) values of 84% at 25 °C and 82% at 35 °C for T3, both of which were higher than the control (50.40% at 25 °C and 35 °C). In contrast, treatments with *Thalassiosira* sp. biomass showed GF% values of 96% for T2 at 25 °C and 90.4% for T1 at 35 °C, both exceeding the control (50% at 25 °C and 35 °C). Additionally, the application of *Thalassiosira* sp. biomass at different concentrations resulted in a 50% increase in the germination index (GI) at 25 °C and a 100% increase at 35 °C. For *N. gaditana* biomass, T3 achieved germination rates of 0.28 and 0.30 germinated seeds h^−1^ at 25 °C and 35 °C, respectively, compared to the control, which reached germination rates of 0.16 and 0.17 seeds h^−1^ at the same temperatures (Table 3). The results for seedling vigor index (SVI) components I, II, and III are presented in Table 4 and Table 5. At 25 °C, T2 in *Thalassiosira* sp. showed significantly higher SVI values across all three components than the control. At 35 °C, T1 and T2 treatments significantly outperformed the control in SVII and SVIII, while only T1 showed a significant increase in SVI (Table 4). Similarly, for *N. gaditana*, T3 exhibited significantly higher SVII and SVIII values at both temperatures than the control, while T1 and T2 showed significantly greater SVI values than the control (Table 5).

### 3.2. Glucose and Amylose Content

The amylose and glucose content in “Higuera” bean seeds is presented in Table 6 and Table 7. The amylose levels align with those reported for Mexican bean varieties, which typically contain 15–20% amylose [45,46]. After germination, the amylose reduction in seeds treated with *Thalassiosira* sp. ranged from 17% (T3) to 54% (T1) at 25 °C, whereas at 35 °C, it fluctuated between 35% and 40% compared to the control, which reduced its initial percentage by 3.5 times (Table 6). Similarly, in seeds treated with *N. gaditana*, the amylose reduction at 25 °C was 15% for the control treatment and 47% for T3, while at 35 °C, T2 and T3 showed reductions of 36–37%, respectively. In contrast, the control and T1 amylose percentage was reduced by 2.5 times with respect to the initial percentage (Table 7).

The initial glucose concentration in bean seeds treated with microalgal extracts from both species showed no significant differences (*p* < 0.05). However, both microalgae species exhibited a similar trend of increased glucose content at both temperatures, with a more significant increase at 35 °C. T3 (*Thalassiosira* sp.) germinated seeds at 25 °C had the highest glucose concentration (1.28 mg·g^−1^ dw), nearly five times higher than before germination. At 35 °C, the glucose concentration in the control was 40% higher than in treatments with *Thalassiosira* sp. (Table 6). For *N. gaditana*, the T3 treatment at 25 °C showed a 30% increase in glucose concentration compared to the control. At 35 °C, seeds treated with *N. gaditana* extracts exhibited a 5- to 10-fold increase in glucose content compared to the control (Table 7).

## 4. Discussion

pH is a crucial factor that directly influences the availability of nutrients in a culture medium, determining the solubility of CO_2_ and minerals and the relative distribution of inorganic carbon species (CO_2_, H_2_CO_3_, HCO_3_^−^, and CO_3_^2−^). This distribution can lead to deficiencies in certain trace elements within the medium. Additionally, the pH of the culture medium affects the dissociation of various salts and complexes. Extreme pH levels can induce toxic or inhibitory effects, ultimately impacting the growth and development of specific microalgae species [47,48].

Previous studies have reported varying P_b_ values for *N. gaditana* under different culture conditions. [49] recorded a maximum P_b_ of 0.4 g·L^−1^·day^−1^ under indoor conditions (20 °C, pH 7.8, irradiance of 300 μE·m^−2^·s^−1^, and D = 0.25 day^−1^). Similarly, [50] obtained a maximum P_b_ of 0.47 g·L^−1^·day^−1^ using a native microalgae mix cultivated in wastewater and tubular photobioreactors under outdoor conditions. According to [16], C_b_ in *N. gaditana* can be increased by at least 300%, reaching a P_b_ of 1.7 g·L^−1^ (0.59 g·L^−1^·day^−1^) at D = 0.34 day^−1^. The optimal conditions selected were pH = 7.5 and D = 0.2 day^−1^ for *Thalassiosira* sp., and D = 0.3 day^−1^ for *N. gaditana*. *Thalassiosira* sp. exhibited higher average C_b_ values of 0.57 and 0.53 g·L^−1^, while *N. gaditana* achieved 0.50–0.52 g·L^−1^ at airflows of 1.0 and 1.5 L·min^−1^, respectively (Figure 4). Agitation is crucial in microalgae cultures, as it directly influences cell growth and development [50]. Additionally, agitation ensures a homogeneous distribution of microalgae and nutrients within the reactor, enhances light absorption, and prevents sedimentation [13]. Five airflow rates (0.5, 1.0, 1.5, 2.0, and 2.5 L·min^−1^) were evaluated to maximize biomass yield during harvesting. Proper agitation control is essential to prevent stagnation and oxygen saturation in the medium and, consequently, the inhibition of photosynthesis and photorespiration in microalgae [14]. For this reason, mass microalgae cultures are typically grown at a much higher CO₂:O₂ ratio than in ambient air.

Germination is a physiological process of seed metabolic reactivation, beginning with the activation stage, which includes imbibition, enzyme synthesis, and cell elongation. This stage is crucial for the seed’s initial condition, as it requires a significant amount of energy in the form of adenosine triphosphate (ATP) [51]. This is followed by a stage of high metabolic activity, where lipids, proteins, and carbohydrates are stored, metabolized, and translocated to the embryo’s growth points [52]. From this point onward, essential compounds required for germination become critical [53,54,55]; many are naturally found in microalgal biomass across different species [56,57]. Both microalgae species in this study exhibited acceptable levels of proteins, carbohydrates, and fatty acids (Table 8), with evidence supporting their role in promoting germination and seedling vigor [57,58].

Both microalgal biomass treatments improved seed performance under all tested conditions. Similar positive effects of microalgal biomass (*Scenedesmus obliquus*), even at low concentrations, have been reported for mung bean and cucumber seeds [59,60].

As mentioned, microalgae synthesize biologically active molecules, including fatty acids, phytohormones, polysaccharides, and phenolic compounds [61]. Ref. [62] reported that *Scenedesmus* spp. and *Arthrospira* spp. contain high concentrations of phytohormones, which promote an increase in dry root weight in ornamental plants. Furthermore, ref. [63] reported that microalgae produce D-amino acids, among which *Thalassiosira* sp. contains D-aspartate and D-alanine. These compounds, either alone or in combination, are involved in stimulating germination rates, speed, and seedling development in *Fabaceae* species. Additionally, certain phytohormones in microalgal biomass directly influence seed germination. Phytohormones regulate various physiological processes in vascular plants, such as growth and development, through a complex signaling network involving key phytohormones such as auxins, cytokinins (CKs), gibberellins (GAs), abscisic acid (ABA), and brassinosteroids (BRs). However, understanding each phytohormone’s specific levels, interactions, and responses to external stimuli remains crucial [64].

Once enough water is absorbed, various metabolites, including starch and glucose, are metabolized during seed germination, playing a central role as an energy source [65]. Enzymes such as α- and β-amylase break down starch (amylose fraction) into sugar molecules (glucose), releasing the energy necessary for seed germination and seedling development [66].

The general trend of amylose reduction and glucose increase, regardless of treatment temperature or microalgae species, follows a logical pattern, as it reflects the conversion of stored reserves from starch (amylose) to sugars (glucose) to generate sufficient energy for germination and embryo growth. However, the rate of amylose decrease was irregular, which could be attributed to the specific components provided by each microalga (Table 4), such as carbohydrates, lipids, and proteins. Being immediately available, these nutrients may have been directly utilized by the seeds, leading to a higher germination rate, a shorter mean germination time, and greater seedling vigor, particularly in the T1 and T2 treatments. However, this trend was not observed in T3, possibly due to the regulation of enzymatic activity caused by an excess of the final product, which may have inhibited further enzymatic conversion [67].

External factors (e.g., temperature fluctuations and chemical imbalances) and internal factors (e.g., seed coat separation) can slow the seed production cycle. Non-germinated seeds or conditions that prolong germination time negatively impact profitability [51]. Evaluating seed germination parameters, such as the germination mean time (GMT), time to 50% germination (T50), the germination frequency (GF%), the germination index (GI), and seedling vigor indices (SVI, SVII, and SVIII), is crucial in assessing seedling quality [22,52]. Reliable and rapid germination is key to efficient crop production [24].

## 5. Conclusions

The cultivation of marine microalgae *Thalassiosira* sp. and *Nannochloropsis gaditana* achieved biomass productivity (P_b_) of 0.145 and 0.147 g·L^−1^·day^−1^, respectively, at a dilution rate of 0.2 day^−1^, an airflow of 0.1 *v*/*v*, and pH 7.0–7.5 and 7.5–8.0, respectively, under laboratory conditions. These strains represent a promising biostimulant alternative to enhance the germination process of agriculturally significant bean seeds (*Phaseolus vulgaris* L.). At doses of 0.5 and 1 g·L^−1^, microalgal biomass significantly stimulated seed germination and seedling vigor in Higuera bean seeds while reducing germination times. This study underscores the biotechnological potential of microalgae as a sustainable biostimulant, demonstrating their ability to enhance seed germination and seedling growth while paving the way for eco-friendly agricultural applications and the development of microalgae-based biofertilizers.

## Figures and Tables

**Table 1:** Composition algae medium.

Macronutrients	Concentration (g L^−1^)
Potassium nitrate (KNO_3_)	100.78
Monobasic sodium phosphate (NaH_2_PO_4_)	6.71
Micronutrients	
Ferric citrate (C_6_H_5_FeO_7_)	2.60
Sodium molybdate (Na_2_MoO_4_)	0.28
Manganese chloride (MnCl_2_)	0.21
Zinc chloride (ZnCl_2_)	0.15
Cobalt chloride (CoCl_2_)	0.02
Copper sulfate (CuSO_4_)	0.03
EDTA (C_10_H_16_N_2_O_8_)	2.35
Vitamins	
Thiamine(C_12_H_17_N_4_OS^+^)	0.26
Biotin (C_10_H_16_N_2_O_3_S)	0.16
Cyanocobalamin (C_63_H_88_CoN_14_O_14_P)	0.2

**Table 2:** Effect of *Thalassiosira* sp. microalgae biomass on the germination of bean seeds under two temperatures (T1 = 0.5 g·L^−1^, T2 = 1.0 g·L^−1^, and T3 = 1.5 g·L^−1^).

Treatments	25 °C				35 °C			
	GMT	T_50_	GF	GI	GMT	T_50_	GF	GI
	(h)	(h)	(%)	(seed·h^−1^)	(h)	(h)	(%)	(seed·h^−1^)
Control	78.37 a	65.23 a	68.80 b	0.22 b	86.55 a	78.39 a	39.20 b	0.09 b
T1	72.79 a	60.06 a	89.60 ab	0.31 ab	71.55 b	67.91 ab	90.40 a	0.32 a
T2	73.11 a	63.12 a	96.00 a	0.33 a	75.95 b	60.28 b	75.20 a	0.25 a
T3	79.53 a	60.40 a	80.80 ab	0.22 b	79.22 ab	64.57 ab	44.80 b	0.14 b

Values followed by an identical lowercase letter in the same column are not significant (*p* ≤ 0.05).

**Table 3:** Effect of *N. gaditana* microalgae biomass on the germination of bean seeds at two temperatures (T1 = 0.5 g·L^−1^, T2 = 1.0 g·L^−1^, and T3 = 1.5 g·L^−1^).

Treatments	25 °C				35 °C			
	GMT	T50	GF	GI	GMT	T50	GF	GI
	(h)	(h)	(%)	(seed·h^−1^)	(h)	(h)	(%)	(seed·h^−1^)
Control	74.34 a	72.87 a	50.40 b	0.16 b	81.66 a	66.17 a	50.40 b	0.17 b
T1	71.92 a	59.78 b	73.67 a	0.26 ab	77.05 ab	69.17 a	69.67 a	0.23 ab
T2	77.52 a	61.98 b	76.00 a	0.25 ab	75.35 ab	66.17 a	75.20 a	0.24 ab
T3	76.50 a	65.21 b	84.80 a	0.28 b	71.13 b	65.21 a	82.40 a	0.29 b

Values followed by an identical lowercase letter in the same column are not significant (*p* ≤ 0.05).

**Table 4:** Effect of *Thalassiosira* sp microalgae biomass on bean seeds’ germination vigor I, II, and III under two temperatures (T1 = 0.5 g·L^−1^, T2 = 1.0 g·L^−1^, and T3 = 1.5 g·L^−1^).

Treatments	25 °C			35 °C		
	Vigor I	Vigor II	Vigor III	Vigor I	Vigor II	Vigor III
Control	0.47 b	14.48 b	7.35 b	0.14 b	8.15 b	4.12 b
T1	1.07 a	18.35 ab	9.50 ab	1.01 a	18.46 a	9.58 a
T2	1.09 a	21.28 a	10.60 a	0.74 ab	16.64 a	8.09 a
T3	0.48 b	18.71 ab	9.04 ab	0.19 b	10.35 b	5.00 b

Values followed by an identical lowercase letter in the same column are not significant (*p* ≤ 0.05).

**Table 5:** Effect of *N. gaditana* microalgae biomass on the vigor I, II, and III of bean seeds under two temperatures (T1 = 0.5 g·L^−1^, T2 = 1.0 g·L^−1^, and T3 = 1.5 g·L^−1^).

Treatments	25 °C			35 °C		
	Vigor I	Vigor II	Vigor III	Vigor I	Vigor II	Vigor III
Control	0.33 b	10.72 b	5.36 b	0.32 c	10.48 b	5.37 b
T1	1.02 a	15.11 ab	7.79 ab	0.66 ab	13.98 ab	7.36 ab
T2	0.84 a	16.91 ab	8.20 ab	0.89 a	15.57 ab	7.61 ab
T3	0.44 b	19.63 a	9.48 a	0.50 bc	19.06 a	9.21 a

Values followed by an identical lowercase letter in the same column are not significant (*p* ≤ 0.05).

**Table 6:** Changes in amylose and glucose content in bean seeds during germination treated with *Thalassiosira* sp microalgae biomass under two temperatures (T1 = 0.5 g·L^−1^, T2 = 1.0 g·L^−1^, and T3 = 1.5 g·L^−1^).

Treatments	Room Temperature		25 °C		35 °C	
	Amylose	Glucose	Amylose	Glucose	Amylose	Glucose
	(%)	(mg·g^−1^ dw)	(%)	(mg·g^−1^ dw)	(%)	(mg·g^−1^ dw)
Control	17.73 ± 0.5 aA	0.66 ± 0.10 aC	9.48 ± 2.6 bB	1.28 ± 0.28 bB	5.16 ± 0.6 bC	3.47 ± 0.27 aA
T1	19.49 ± 0.9 aA	0.61 ± 0.02 aC	8.88 ± 1.6 bB	1.01 ± 0.02 bB	11.69 ± 1.0 aB	2.28 ± 0.16 bA
T2	18.73 ± 0.6 aA	0.68 ± 0.81 aC	8.99 ± 0.6 bB	1.56 ± 0.02 bB	12.15 ± 0.7 aB	2.40 ± 0.15 bA
T3	18.72 ± 1.5 aA	0.56 ± 0.10 aC	15.53 ± 2.6 aA	2.68 ± 0.25 aA	12.21 ± 1.2 aB	1.11 ± 0.21 cB

Values followed by an identical lowercase letter in the same column and a capital letter in the same row and column of the same variable are not significant (*p* ≤ 0.05).

**Table 7:** Changes in amylose and glucose content in bean seeds during germination treated with *N. gaditana* microalgae biomass under two temperatures (T1 = 0.5 g·L^−1^, T2 = 1.0 g·L^−1^, and T3 = 1.5 g·L^−1^).

Treatments	Room Temperature		25 °C		35 °C	
	Amylose	Glucose	Amylose	Glucose	Amylose	Glucose
	(%)	(mg·g^−1^ dw)	(%)	(mg·g^−1^ dw)	(%)	(mg·g^−1^ dw)
Control	17.73 ± 1.4 aA	0.66 ± 0.10 aB	15.08 ± 0.4 aB	0.91 ± 0.01 bA	7.24 ± 0.7 cC	0.55 ± 0.04 cB
T1	16.24 ± 0.4 aA	0.64 ± 0.02 aB	12.17 ± 1.8 abB	0.66 ± 0.01 bB	6.39 ± 0.4 dC	5.14 ± 0.10 aA
T2	15.31 ± 1.8 aA	0.53 ± 0.31 aB	11.69 ± 1.2 abB	0.89 ± 0.23 bB	9.60 ± 0.9 bB	2.62 ± 0.29 bA
T3	17.69 ± 1.8 aA	0.56 ± 0.10 aC	9.33 ± 2.9 bB	1.28 ± 0.25 aB	11.15 ± 0.1 aB	2.47 ± 0.03 bA

Values followed by an identical lowercase letter in the same column and a capital letter in the same row and column of the same variable are not significant (*p* ≤ 0.05).

**Table 8:** Biomass composition of the microalgae *Nannochloropsis gaditana* and *Thalassiosira* sp. on a dry basis.

Composition (%)	*Nannochloropsis gaditana*	*Thalassiosira* sp.
Proteins	62.16 ± 0.063	60.15 ± 0.060
Lipids	14.80 ± 0.010	24.02 ± 0.002
Carbohydrates	11.98 ± 0.025	4.32 ± 0.022
Ashes	11.06 ± 0.001	11.51 ± 0.005

## Data Availability

Data are available to be requested from the author through correspondence.
